# Qingfei Tongluo Formula Mitigates *Mycoplasma pneumoniae* Infection via the PERK Signaling Pathway

**DOI:** 10.1155/2022/9340353

**Published:** 2022-12-06

**Authors:** Xiuxiu Liu, Mingjing Wang, Qianna Kan, Yan Lin, Zhiyan Jiang

**Affiliations:** Department of Pediatrics, Longhua Hospital Affiliated to Shanghai University of Traditional Chinese Medicine, China

## Abstract

*Mycoplasma pneumoniae* pneumonia (MPP) is usually found in school-aged children and relapses easily because of antibiotic resistance. The Qingfei Tongluo formula (QTF) is a clinically used traditional Chinese medicine to treat MPP. Our previous study demonstrated that QTF exhibited ameliorative effects on the experimental MPP mice model. In this study, the function and underlying QTF mechanism in MPP was attempted to be further explored. Mycoplasma pneumoniae (MP) was applied to infect A549 cells and BALB/c mice to mimic MPP *in vitro* and *in vivo*. Cytokine release and reactive oxygen species (ROS) production were analyzed using enzyme-linked immunosorbent assay (ELISA) assay and flow cytometry. Western blot analysis was used to detect the protein involved in ER stress. MP infection was found to enhance cytokine release and ER stress *in vitro* and *in vivo*, and this effect could be alleviated by QTF. Moreover, protein kinase RNA-like endoplasmic reticulum kinase (PERK) knockdown alleviated MP infection-induced cytokine release, ROS production, and ER stress in A549 cells while the PERK overexpression exhibited the opposite effects. In conclusion, QTF alleviated MP infection-induced cytokine release, ROS production, and ER stress via PERK signaling pathway inhibition.

## 1. Introduction

Respiratory tract infection has been, and remains to be, a major public health concern, which can range in severity from mild to life-threatening, and is especially dangerous in children [1]. *Mycoplasma pneumoniae* (MP), a common bacterial pathogen, is considered as an important respiratory tract infection cause [2]. MP is also associated with community-based pneumonia, particularly among school-aged children and young adults. MP-induced pneumonia (MPP) easily relapses due to antibiotic resistance. Therefore, new and more effective drugs are needed. Traditional Chinese medicine attracted the researchers' attention due to its increased safety and effectiveness.

The traditional Chinese medicine Qingfei Tongluo formula (QTF) was developed by pediatricians at the Longhua Hospital (Shanghai, China). It has been widely used to treat MPP with azithromycin (AZM) in clinics showing a good curative effect. QTF is composed of the following Chinese medicines: cortex mori from *Morus alba* L. (12% *w*/*w*), cortex lycii from *Lycium chinense Mill*. (12% *w*/*w*), peach kernel from *Prunus persica* L. (12% *w*/*w*), aidicha from *Ardisia japonica* (Thumb.) Blume (12% *w*/*w*), lumbricus from *Pheretimaas pergilum* (E Perrier) (12% *w*/*w*), almond from *Amygdalus communis* Vas (12% *w*/*w*), seed from *Perilla frutescens* (L.) Britt. (12% *w*/*w*), semen lepidii from *Heleocharis dulcis* (Burm. f.) Trin. (12% *w*/*w*), and licorice from *Glycyrrhiza uralensis* Fisch. (4% *w*/*w*). Our previous study reported that QTF exhibited ameliorative effects on the *MP* mice model [3]. Furthermore, this study demonstrated that QTF treatment showed better ameliorative effects than azithromycin in MPP patients [4]. An important component of QTF, naringenin, suppressed the inflammatory response and pulmonary fibrosis by autophagy inhibition after MP infection [5]. In addition, QTF treatment has a significant effect on children recovering from pneumonia based on conventional Western medicine treatment [6]. However, the underlying mechanism remains largely unknown.

The endoplasmic reticulum (ER) is an important organelle in eukaryotic cells, which is responsible for the synthesis and folding of proteins and storage of free calcium [7]. Some physiological stress including Ca^2+^ levels, disturbances in redox, or other environmental factors induce misfolded protein accumulation leading to ER stress. Triggering the unfolded protein response (UPR), the ER stress mainly involves three signaling pathways: inositol-requiring enzyme-1*α* (IRE1*α*), activating transcription factor 6 (ATF6), and protein kinase RNA-like endoplasmic reticulum kinase (PERK) [8]. UPR is also associated with several major cellular activities including proinflammatory response, autophagy, apoptosis, innate immunity, and the mitogen-activated protein kinase pathways [9].

In the current study, QTF was applied to treat MP infected A549 cells and BALB/c mice. The results indicated that QTF played a protective role in MP infection *in vitro* and *in vivo*.

## 2. Methods

### 2.1. MP Culture

MP, ATCC15531 strain (American Type Culture Collection, Rockville, MD, USA), was cultured in modified Hayflick medium (GZBIOTEST Co., Ltd, Guangdong, China) supplemented with horse serum, PPLO broth, 25% yeast extract, 0.5% glucose, 0.025% thallium acetate, penicillin G (1000 U/mL), and 0.002% phenol red, pH 7.6, at 37°C, 5% CO_2_ for 7 days.

### 2.2. Cell Culture and Infection

A549 and 293 T cells were purchased from the Shanghai Cell Bank, Chinese Academy of Sciences (Shanghai, China) and cultured in DMEM (Gibco, Grand Island, NY, USA) containing 10% fetal bovine serum (Gibco) at 37°C, 5% CO_2_.

For MP infection, the culture medium was replaced with MP infection medium containing 10% MP (approximately 1 × 10^7^ CFU/10^6^ cells), unless otherwise specified, and cultured for 12, 24, and 48 h. Culture medium containing only mycoplasma broth without bacteria was used to culture control cells.

### 2.3. ELISA

The ELISA kit (Jiancheng, Nanjing, China) was used to determine the IL-8 and TNF-*α* contents according to the manufacturer's protocol.

### 2.4. Real-Time PCR

Total RNA was extracted using TRIzol Reagent (Invitrogen, Carlsbad, CA, USA) following the manufacturer's instructions. Total RNA one mcg was used to generate cDNAs using the Revert AidTM First Strand cDNA Synthesis Kit (#K1622; Thermo Fisher Scientific Inc., Grand Island, NY, USA) with random primer. Real-time PCR was then performed to quantify mRNA levels with SYBR Green PCR Master Mix (#K0223; Thermo) on an ABI 7300 thermocycler (Applied Biosystems, Foster City, CA, USA). The 2^−*Δ*Ct^ method was used to quantify relative gene expression levels, and *β*-actin was used as an internal control. The primers were as follows:

PERKPrimer F 5′ATCGCAGAGGCAGTGGAG 3′

Primer R 5′CATTGGGGTCAGAACCGT 3′

IRE1*αα*Primer F 5′GAGGACAGGCTCAATCAA 3′

Primer R 5′GGTCAGGAGGTCAATAACA 3′

ATF6Primer F 5′TTCCGTGACTAAACCTGT 3′

Primer R 5′TTAATCTCGCCTCTAACC 3′

*β*-ActinPrimer F 5′TGGCATTGCCGACAGG 3′

Primer R 5′GCATTTGCGGTGGACG 3′

### 2.5. QTF Extract

All TCM were purchased from Shanghai Huayu Chinese Herbs Co., Ltd. including bark of *Morus alba* L. (XD14122403, Henan Province), bark of *Lycium Chinese Mill*. (LY1501125, Shanxi Province), peach kernel from *Prunus persica L*. (XD14082202, Hebei Province), whole plant of *Ardisia japonica* (Thumb.) Blume (LY1410211, Henan Province), *Pheretimaas pergilum* (E Perrier) (LY1412060, Shanghai City), almond from *Amygdalus communis* Vas (XD14071106, Hebei Province), seed of *Perilla frutescens* (L.) Britt. (YT14121701, Gansu Province), *Heleocharis dulcis* (Burm. f.) Trin. (DH14092405, Shandong Province), and rhizome of *Glycyrrhiza uralensis* Fisch. (HY14112503, Gansu Province). The QTF components (250 g) were pulverized with a motor-driven grinder to prepare the extract. The extract was then refluxed twice with distilled water (2 L) for 1 h each time, followed by filtration, centrifugation, and evaporation to dryness under reduced pressure in a rotary evaporator with a yield of 13.6%.

### 2.6. Evaluation of Reactive Oxygen Species (ROS)

Cells were probed with 10 *μ*M of 2′,7′-dichlorodihydrofluorescein diacetate (DCFH-DA) (Beyotime, Shanghai, China) at 37°C for 20 min, washed with DMEM (without FBS), and harvested using trypsin. ROS level was analyzed using a flow cytometry (BD Biosciences, Franklin Lakes, NJ, USA).

### 2.7. Western Blot

Total cells and tissues were prepared with RIPA buffer containing proteinase inhibitor (Pierce, Rockford, IL, USA). NE-PER™ Nuclear and Cytoplasmic Extraction Reagents (Thermo Fisher Scientific) were used to separate the cytosolic fraction and nuclear extracts. Supernatant samples were separated by 10% or 15% SDS-PAGE and transferred onto nitrocellulose membranes (Millipore, Billerica, WI, USA). The membranes were probed, after blocking with 5% nonfat milk, with primary antibodies (anti-PERK, Abcam, Ab229912; anti-eIF2, Abcam, Ab169528; anti p-eIF2, Abcam, Ab32157; anti-ATF4, Proteintech, 10835-1-AP; anti-CHOP, Proteintech, 15204-1-AP; anti-*β*-actin, Proteintech, 66009-1-lg; anti-NF-*κ*B, Abcam, Ab16502; and anti-H3, Proteintech, 17168-1-AP) at 4°C overnight. After washing away the unbound antibody on the second day, the membranes were washed and incubated with HRP-conjugated rabbit secondary antibody (Beyotime) at room temperature for 1 h. The signals from the immunoreactive proteins were detected using the ECL reagent (Millipore).

### 2.8. Lentivirus Preparation

Short hairpin RNA (shRNA) oligos targeting PERK (shPERK-1: 5′-GCACUUUAGAUGGGAGAAUTT-3′, site: 516-534; shPERK-2: 5′-GCAUGGAAACAGUUCCUUUTT-3′, site: 699-717; and shPERK-3: 5′-GCAUCUGCCUGGUUACUUATT-3′, site: 1208-1226), along with a negative control shNC (5′-UUCUCCGAACGUGUCACGUTT-3′), were annealed and cloned into AgeI- and EcoRI-digested pLKO.1 (Addgene, Cambridge, MA, USA). The full-length human PERK was cloned into pLVX-puro lentiviral plasmid (Clontech, Palo Alto, CA, USA). The 293 T cells were cotransfected with pLKO.1-shPERK or pLVX-PERK plasmid (20 *μ*g) and packaging plasmids, psPAX2 and pMD2.G, using Lipofectamine 2000 (Invitrogen) to generate the lentivirus. After 48 h, the supernatant was harvested, and the viral titer was calculated by transducing the 293 T cells.

### 2.9. TUNEL Staining

Treated cells were cultured overnight. After rising twice with PBS, the cells were fixed with 4% paraformaldehyde for 15 min and permeabilized in 0.25% Triton X-100 for 20 min. The cells were first incubated in terminal dexynucleotidyl transferase reaction cocktail for 45 min at 37°C, followed by treatment with Click-iT reaction cocktail. The 4′-6-diamidino-2-phenylindole (DAPI) (Beyotime Biotech) was used to stain the nuclei.

### 2.10. Immunofluorescence

Treated cells were cultured overnight. The cells were fixed with 4% paraformaldehyde for 15 min after rising twice with PBS. The cells were then subjected to immunofluorescence staining with anti-caspase 3 (Abcam, ab32351), anti-PERK (Abcam, ab65142), and anti-CHOP (Abcam, ab11419), respectively, at 4°C overnight, followed by staining with Alexa Fluor 488 linked secondary antibody. DAPI was used to stain the nuclei.

### 2.11. MP Infection Mouse Model

The 4-week-old BALB/c mice (15 ± 1 g) were obtained from Shanghai Sippr-Bk Laboratory Animals Co., Ltd. (Shanghai, China). All mice were randomly divided into five groups: control, model, QTF low dose, QTF middle dose, and QTF high dose (*n* = 6). Control group mice were treated with 100 *μ*L normal saline by nasal drip. All the other groups were treated with nasal drops containing 100 *μ*L MPFH (1 × 10^7^ ccu/mL) for 2 days. A 0.25 mL/20 g body weight normal saline was orally given to the mice in the control and model groups by gavage once a day. QTF groups were orally given 0.425, 0.85, and 1.7 g/kg QTF for three consecutive days, respectively. Ethical approval for the study was provided by the Independent Ethics Committee of Shanghai University of Traditional Chinese Medicine. The right lung inferior lobe was harvested on the fifth day and fixed with 4% paraformaldehyde, embedded with paraffin, and cut into slices for hematoxylin and eosin staining. An optical microscope (Leica DMLB, Germany) was used to perform morphometric analysis. Alveolar lavage fluid was harvested to detect the release of IL-8 and TNF-*α*.

### 2.12. Statistical Analysis

All the experiments were conducted at least three times. Data were expressed as means ± standard deviations. The ANOVA test was used to perform statistical analysis. A *P* value of <0.05 indicated a significant difference.

## 3. Results

### 3.1. MP Infection Induces Cytokine Expression and ER Stress

**Figure 1:**
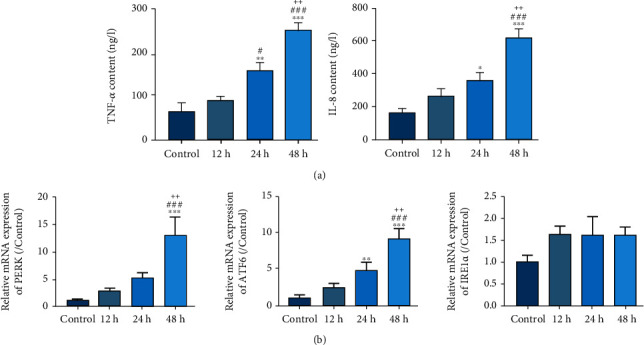
MP infection induces cytokine expression and ER stress. (a) TNF-*α* and IL-8 production were measured using ELISA assay after MP treatment (10^7^ CFU/10^6^ cells) for 12, 24, and 48 h in A549 cells. (b) PERK, ATF6, and IRE1*α* mRNA levels were measured using real-time PCR. ^∗^*P* < 0.05, ^∗∗^*P* < 0.01, and ^∗∗∗^*P* < 0.001 vs. control; #*P* < 0.05, ###*P* < 0.001 vs. 12 h; ++*P* < 0.01 vs. 24 h.

MP was applied to A549 cells and cultured for 12, 24, and 48 h. MP infection increased the production of TNF-*α* and IL-8 in a time-dependent manner as shown in Figure 1(a). ATF6, PERK, and IRE1*α* are the three major signaling pathways involved in ER stress. Therefore, the levels of ATF6, PERK, and IRE1*α* were detected using real-time PCR. The results illustrated significantly upregulated PERK and ATF6 mRNA levels upon MP infection. However, there was no significant change in IRE1*α* level (Figure 1(b)), indicating that IRE1*α* signaling pathway may not be affected by MP infection.

### 3.2. QTF Alleviates MP Infection-Induced Cytokine Release, ROS Production, and ER Stress in A549 Cells

**Figure 2:**
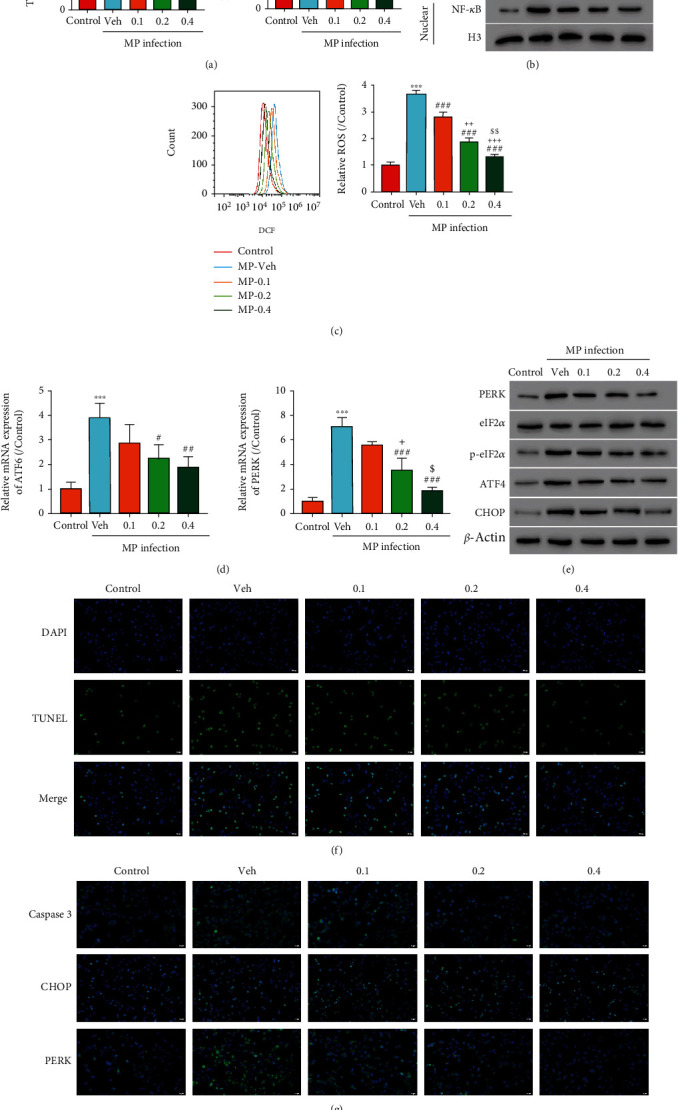
QTF alleviates MP infection-induced cytokine release, ROS production, and ER stress in A549 cells. (a) The TNF-*α* and IL-8 release. (b) Western blot assay was used to detect the cytoplasmic and nuclear NF-*κ*B protein levels. (c) Flow cytometry was used to measure ROS production. (d) Real-time PCR was used to measure PERK and ATF6 mRNA levels. (e) The PERK, eIF2*α*, p-eIF2*α*, ATF4, and CHOP protein levels were measured. (f) TUNEL assay was performed to detect apoptotic cells. ×200. (g) Immunofluorescence was used to measure caspase 3, CHOP, and PERK levels. ×200. ^∗∗∗^*P* < 0.001 vs. control; #*P* < 0.05, ##*P* < 0.01, and ###*P* < 0.001 vs. vehicle; +*P* < 0.05, ++*P* < 0.01, and +++*P* < 0.001 vs. 0.1 mg/mL; $*P* < 0.05, $$*P* < 0.01 vs 0.2 mg/mL.

Different QTF concentrations (0, 0.1, 0.2, and 0.4 mg/mL) were applied to MP infected A549 cells to explore QTF function. ELISA assay showed that the IL-8 and TNF-*α* increase induced by MP was inhibited after MP treatment in a dose-dependent manner (Figure 2(a)). Nuclear factor-*κ*B (NF-*κ*B) is a common anti-inflammatory target that has been related to proinflammatory cytokine production, such as TNF-*α* and IL-8 [10]. The nuclear NF-*κ*B level was promoted after MP infection as shown in Figure 2(b) but depressed upon QTF treatment. Opposite effects were observed of the cytoplasmic NF-*κ*B level, indicating that MP induced the NF-*κ*B activation which could be inhibited by QTF. Furthermore, ROS has been reported to regulate NF-*κ*B activity, so we detected ROS level upon treatment with MP and QTF. MP infection increased the ROS level in A549 cells as expected; however, cells treated with MP and QTF exhibited lower ROS level than the cells treated with MP only (Figure 2(c)). In addition, the increase PERK and ATF6 levels induced by MP were alleviated after QTF treatment (Figure 2(d)), suggesting that PERK and ATF6 signaling pathways were involved in QTF function in MP-treated cells. MP infection upregulated the protein levels of PERK, p-eIF2*α*, ATF4, and CHOP as shown in Figure 2(e). QTF could decrease these protein levels in a dose-dependent manner. The TUNEL results showed that MP infection increased the apoptotic cell number, and QTF reduced the apoptotic cell number (Figure 2(f)). MP infection enhanced caspase 3, CHOP, and PERK expression according to the immunofluorescence (Figure 2(g)), while QTF alleviated these effects. Taken together, QTF mitigated the effects induced by MP infection in A549 cells.

### 3.3. PERK Silencing Alleviates MP Infection-Induced Cytokine Release, ROS Production, and ER Stress in A549 Cells

**Figure 3:**
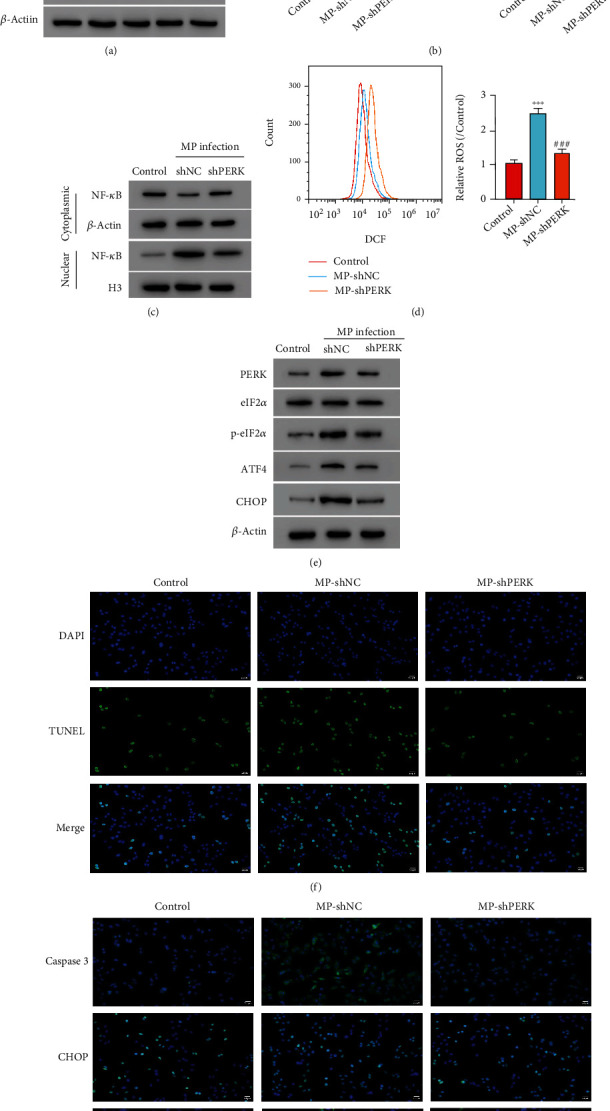
PERK knockdown alleviates MP infection-induced cytokine release, ROS production, and ER stress in A549 cells. (a) The PERK protein level was detected after shPERK lentivirus transduction. (b) TNF-*α* and IL-8 release. (c) Western blot assay was used to detect the cytoplasmic and nuclear NF-*κ*B protein levels. (d) ROS production was measured by flow cytometry. (e) The PERK, eIF2*α*, p-eIF2*α*, ATF4, and CHOP protein levels were measured. (f) TUNEL assay was performed to detect apoptotic cells. ×200. (g) The caspase 3, CHOP, and PERK levels were measured using immunofluorescence. ×200. ^∗∗^*P* < 0.01, ^∗∗∗^*P* < 0.001 vs. control; #*P* < 0.05, ##*P* < 0.01, and ###*P* < 0.001 vs. MP-shNC.

Knockdown lentiviruses targeting PERK were applied to A549 cells upon MP infection to confirm whether PERK was involved in MP infection. The three shPERK lentiviruses significantly decreased the protein PERK level in A549 cells as shown in Figure 3(a). The shPERK-2 gives the best silencing effect. Thus, shPERK-2 lentivirus was used in the following studies to knockdown PERK level. Furthermore, this study found that PERK silencing could alleviate MP infection-induced TNF-*α* and IL-8 release (Figure 3(b)), NF-*κ*B activation (Figure 3(c)), and ROS production (Figure 3(d)). In addition, the PERK, p-eIF2*α*, ATF4, and CHOP level increases induced by MP infection were inhibited by PERK silencing (Figure 3(e)). TUNEL results showed that PERK silencing reduced the increased apoptotic cell number induced by MP infection (Figure 3(f)). According to the immunofluorescence (Figure 3(g)), MP infection enhanced the caspase 3, CHOP, and PERK expression, while PERK silencing alleviated these effects. These results further confirmed that PERK signaling pathway played a role in MP infection.

### 3.4. QTF Alleviates PERK Overexpression-Induced Cytokine Release, ROS Production, and ER Stress in A549 Cells

**Figure 4:**
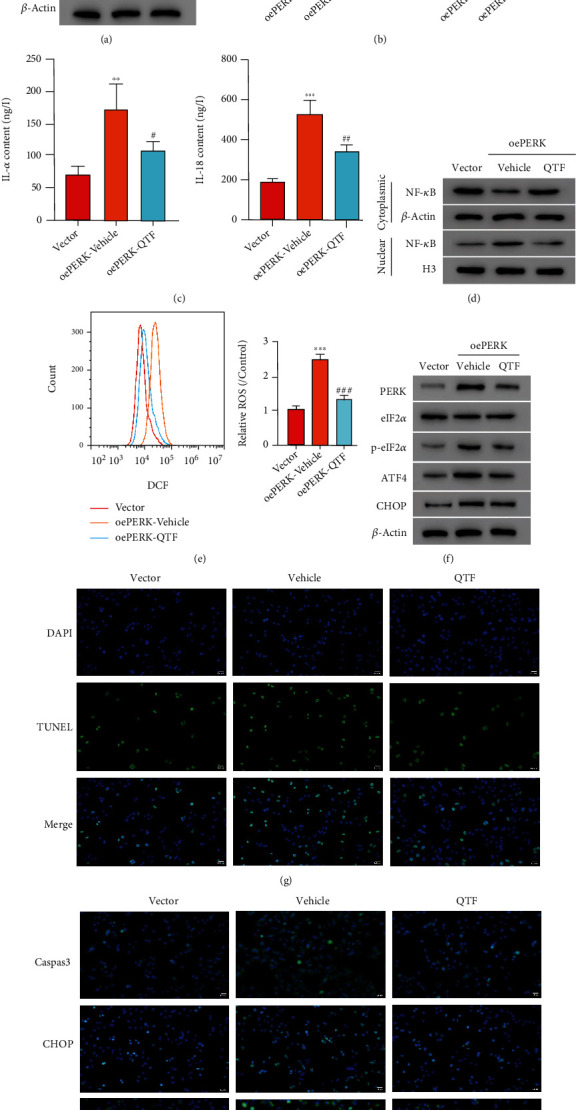
QTF mitigates PERK overexpression-induced cytokine release, ROS production, and ER stress in A549 cells. (a) Protein PERK level was detected after transduction of PERK overexpressing lentivirus. (b) A549 cells were treated with PERK overexpressing lentivirus and/or 10 *μ*M PDTC. ELISA assay was used to detect the TNF-*α* and IL-8 release. (c–f) A549 cells were treated with PERK overexpressing lentivirus and/or 0.2 mg/mL QTF. The TNF-*α* and IL-8 release (c), NF-*Κ*b activation (d), ROS production (e), and the PERK, eIF2*α*, p-eIF2*α*, ATF4, and CHOP protein levels (f) were measured. (g) TUNEL assay was performed to detect apoptotic cells. ×200. (H) The caspase 3, CHOP, and PERK levels were measured using immunofluorescence. ×200. ^∗∗^*P* < 0.01, ^∗∗∗^*P* < 0.001 vs. vector; #*P* < 0.05, ##*P* < 0.01, and ###*P* < 0.001 vs. oePERK+vehicle.

PERK overexpressing lentivirus was used to enhance the PERK expression. As expected, the PERK protein level was markedly upregulated by PERK overexpressing lentivirus (Figure 4(a)). ShPERK depressed MP infection-induced activation of NF-*κ*B based on the results in Figure 3(c). PDTC, an NF-*κ*B inhibitor, was applied to A549 cell. The PERK overexpression promoted the TNF-*α* and IL-8 release as shown in Figure 4(b); however, cotreatment with PERK overexpressing lentivirus and PDTC showed lower TNF-*α* and IL-8 levels than that in the cells treated with PERK overexpressing lentivirus alone. Moreover, the PERK overexpression could increase the TNF-*α* and IL-8 release (Figure 4(c)), NF-*κ*B activation (Figure 4(d)), ROS production (Figure 4(e)), and the PERK, p-eIF2*α*, ATF4, and CHOP (Figure 4(f)) protein levels. These effects were mitigated by QTF in A549 cells. The TUNEL results showed that the PERK overexpression induced apoptotic cell increase, and that QTF could reduce the apoptotic cell number (Figure 4(g)). QTF could decrease the enhanced caspase 3, CHOP, and PERK expressions induced by the PERK overexpression. In summary, these results indicated that QTF played a protective role in the A549 cell via PERK signaling pathways.

### 3.5. QTF Mitigates MP Infection-Induced Cytokine Release and ER Stress *In Vivo*

**Figure 5:**
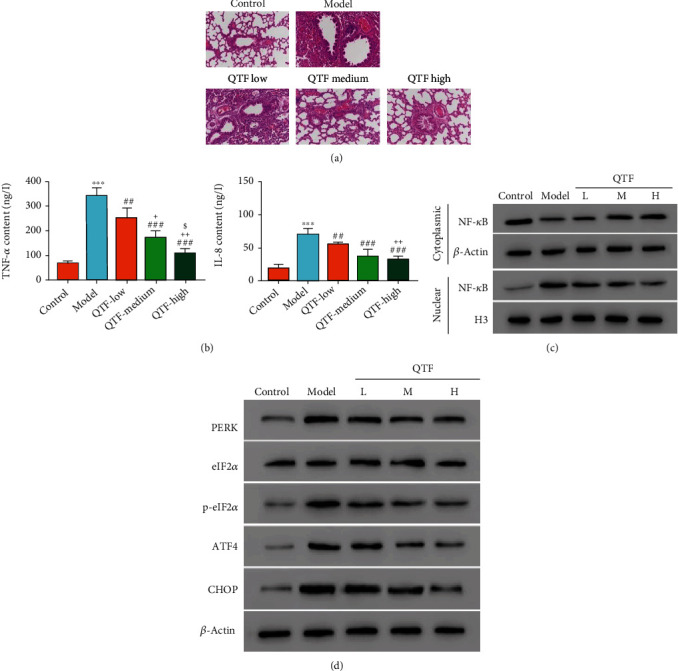
QTF mitigates MP infection-induced cytokine release and ER stress *in vivo*. (a) Lung tissue HE staining from each group. ×200. (b) The TNF-*α* and IL-8 release. (c) Western blot assay was used to detect the protein levels of cytoplasmic and nuclear NF-*κ*B. (d) The protein PERK, eIF2*α*, p-eIF2*α*, ATF4, and CHOP levels were measured. ^∗∗∗^*P* < 0.001 vs. control; ##*P* < 0.01, ###*P* < 0.001 vs. model; +*P* < 0.05, ++*P* < 0.01 vs. QTF low dose; $*P* < 0.05 vs. QTF-medium dose.

The MP infection mouse model was established and treated with low, middle, and high dose of QTF to further explore QTF function. HE staining showed that relatively normal lung tissues were observed in the control group. There were many pulmonary interstitial infiltrations of lymphocytes and plasmacytes, as well as bronchus and vasodilation congestion in the model group. QTF relieved the injury induced by MP infection in a dose-dependent manner (Figure 5(a)). Furthermore, this study found that QTF alleviated MP infection which induced the release of TNF-*α* and IL-8 (Figure 5(b)), NF-*κ*B activation (Figure 5(c)), and the protein levels of PERK, p-eIF2*α*, ATF4, and CHOP (Figure 5(d)) in vivo, indicating that QTF played a protective role in the MP infection mouse model.

## 4. Discussion

MP infection is the cause of several diseases including pneumonia. The MPP incidence in children is increasing [11]. Researchers prefer to focus on exploring the mechanism under antibiotic MPP treatment. The effects of traditional Chinese medicine were usually ignored. QTF was initially developed by the Pediatric Department at Shanghai Longhua Hospital and exhibited an effective role in the treatment of children with MPP [12]. Our previous study found that QTF showed ameliorative effects on the MPP mice model [3]. This study demonstrated that QTF alleviated MP infection-induced cytokine release, ROS production, and ER stress *in vitro* and *in vivo*.

When infection occurs, our body's first response should be to the inflammation caused by the innate immunity. TNF-*α* and IL-8 are the two important cytokines of the inflammation process. TNF-*α*, which is produced by monocytes and macrophages, plays a key role in MP infection-induced lung injury [13]. Higher TNF-*α* serum level is observed in children with MPP [14]. Recently, high expressions of both TNF-*α* and community-acquired respiratory distress syndrome toxins are considered to be a good predictor for MPP [15]. MP has been found to induce IL-8 production in bronchial epithelial cells via NF-*κ*B or ERK signaling pathway [16]. This current study found that MP infection enhanced the TNF-*α* and IL-8 release *in vitro* and *in vivo*, and QTF could decrease TNF-*α* and IL-8 levels. In addition, MP infection was also observed to increase ROS generation, and this finding was consistent with Sun et al. [17].

A growing number of researchers demonstrate that ER stress and sustained UPR signaling contribute significantly to viral infections and inflammatory disorders [18]. Viruses may interact with the host UPR and the release of virions that generate abundant unfolded or misfolded proteins causing ER stress [19]. Chlamydia pneumoniae infection has been found to induce ER stress/UPR, leading to increased ROS and cytosolic Ca^2+^ levels [20]. It is well documented that ER stress is strongly associated with pneumonia, such as COVID-19 [9]. EIF2 phosphorylation, ATF4, and CHOP upregulation are observed after SARS-CoV infection, resulting in PERK activation [21]. In the current study, PERK signaling pathway was activated by MP infection, and QTF could block that, suggesting that QTF reduced MP infection-induced ER stress via PERK signaling pathways. Moreover, the NF-*κ*B pathway activation caused by the infection of many bacterial pathogens through induction of the host UPR has been well studied [22, 23]. NF-*κ*B activation after MP infection in this study was also found, suggesting that MP enhanced the NF-*κ*B pathway through increase of the ER stress by upregulating PERK.

## 5. Conclusions

**Figure 6:**
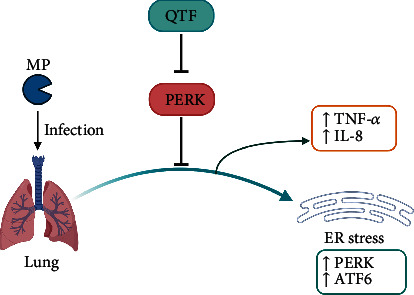
QTF mitigates MP infection via PERK signaling pathway.

QTF alleviated MP-induced cytokine release, ROS production, and ER stress via PERK signaling pathway inhibition (Figure 6).

## Data Availability

The datasets generated and/or analyzed during the current study are available from the corresponding author on reasonable request.
